# Killian‐Jamieson diverticulum mimicking a suspicious thyroid lesion

**DOI:** 10.1002/ccr3.1833

**Published:** 2018-10-31

**Authors:** Willian Schmitt, Ana Germano

**Affiliations:** ^1^ Department of Radiology Hospital Prof. Doutor Fernando Fonseca Amadora Portugal

**Keywords:** barium esophagogram, biopsy, fine‐needle, diverticulum, thyroid nodule, ultrasound

## Abstract

Killian‐Jamieson diverticulum represents a rare form of esophageal diverticulum originating on the anterolateral wall of the cervical esophagus. Despite its rarity, it is crucial to recognize this entity, with such specific imaging findings, to avoid unnecessary invasive procedures such as fine‐needle aspiration or even surgery.

A 77‐year‐old male patient presented with a 1‐year history of swallowing foreign body sensation.

He denied dyspnea, dysphagia, or dysphonia. No other ENT symptoms were specified.

Physical exam and laboratory blood tests were unremarkable.

A neck ultrasound performed in another institution identified a suspicious left lobe hypoechoic thyroid nodule, containing microcalcifications. The patient was referred for fine‐needle aspiration biopsy (FNA).

The ultrasound (US) examination performed prior to FNA, revealed a communication of this nodule with the posteriorly located esophagus (Figure 1). Passage of air and saliva from the esophagus to the nodule was demonstrated during swallowing (Video S1). The diagnosis of Killian‐Jamieson diverticulum was made by US and posteriorly confirmed by a barium swallowing pharyngoesophagography (Figure 2). As so, requested FNA was not performed.

**Figure 1:**
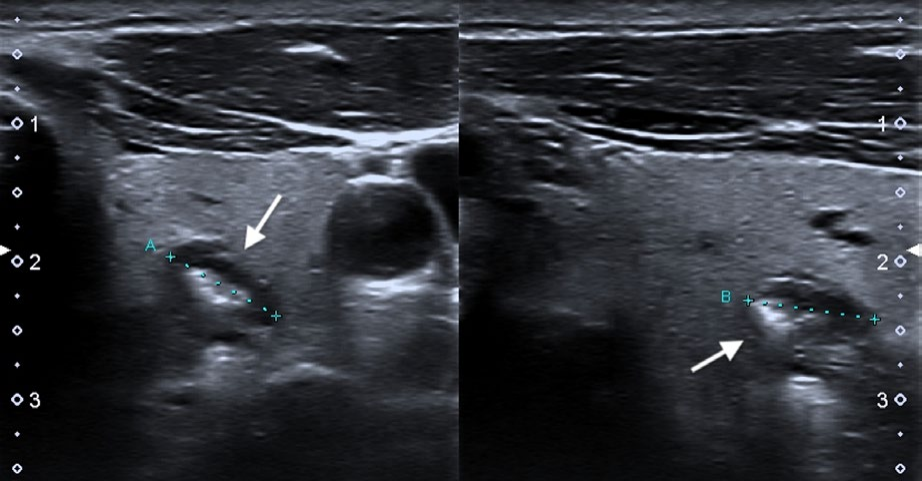
Thyroid ultrasound examination showed a hypoechoic nodule with bright internal hyperechoic foci and a partial surrounding halo involving the posterior aspect of the left thyroid lobe (white arrow)

**Figure 2:**
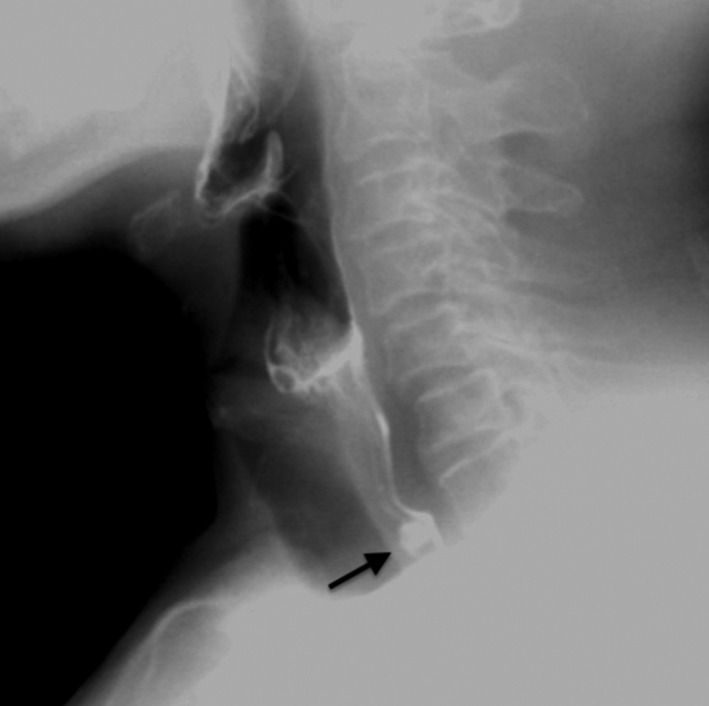
Barium swallow study depicted a left barium‐filled sac protruding from the left anterolateral wall of the cervical esophagus (black arrow)

No further treatment was implemented and the patient is stable at six‐month follow‐up.

Killian‐Jamieson diverticulum represents a rare entity, originating on the anterolateral wall of the cervical esophagus, below the cricopharyngeal muscle. This diagnosis should be considered when a thyroid nodule is located near the esophagus, especially when it ultrasound features changes after swallowing.^1^

Despite its rarity, it is crucial to recognize this entity, with such specific imaging findings, to avoid unnecessary invasive procedures such as FNA or even surgery.^2^

## AUTHOR CONTRIBUTION

WS: Contributed to write the case and identify the images. AG: Reviewed and edited the case report and helped in identifying appropriate images.
